# Spoken Language Development and the Challenge of Skill Integration

**DOI:** 10.3389/fpsyg.2019.02777

**Published:** 2019-12-17

**Authors:** Aude Noiray, Anisia Popescu, Helene Killmer, Elina Rubertus, Stella Krüger, Lisa Hintermeier

**Affiliations:** ^1^Laboratory for Oral Language Acquisition, Linguistic Department, University of Potsdam, Potsdam, Germany; ^2^Haskins Laboratories, New Haven, CT, United States; ^3^Department of Linguistics, University of Oslo, Oslo, Norway; ^4^Department of Education, Jyväskylä University, Jyväskylä, Finland

**Keywords:** language acquisition, coarticulation, speech motor control, phonological awareness, vocabulary, speech production

## Abstract

The development of phonological awareness, the knowledge of the structural combinatoriality of a language, has been widely investigated in relation to reading (dis)ability across languages. However, the extent to which knowledge of phonemic units may interact with spoken language organization in (transparent) alphabetical languages has hardly been investigated. The present study examined whether phonemic awareness correlates with coarticulation degree, commonly used as a metric for estimating the size of children’s production units. A speech production task was designed to test for developmental differences in intra-syllabic coarticulation degree in 41 German children from 4 to 7 years of age. The technique of ultrasound imaging allowed for comparing the articulatory foundations of children’s coarticulatory patterns. Four behavioral tasks assessing various levels of phonological awareness from large to small units and expressive vocabulary were also administered. Generalized additive modeling revealed strong interactions between children’s vocabulary and phonological awareness with coarticulatory patterns. Greater knowledge of sub-lexical units was associated with lower intra-syllabic coarticulation degree and greater differentiation of articulatory gestures for individual segments. This interaction was mostly nonlinear: an increase in children’s phonological proficiency was not systematically associated with an equivalent change in coarticulation degree. Similar findings were drawn between vocabulary and coarticulatory patterns. Overall, results suggest that the process of developing spoken language fluency involves dynamical interactions between cognitive and speech motor domains. Arguments for an integrated-interactive approach to skill development are discussed.

## Introduction

In the first decade of life, most children learn to speak their native language effortlessly, without explicit instruction but with daily exposure and experiencing of their native language as a speech motor activity. With the gradual expansion of children’s expressive repertoire comes the fine tuning of phonological knowledge (e.g., Ferguson and Farwell, 1975; Menn and Butterworth, 1983; Beckman and Edwards, 2000; Munson et al., 2012). While relationships between lexical and phonological developments have been well documented over the last decades (Storkel and Morrisette, 2002; Edwards et al., 2004, 2011; Stoel-Gammon, 2011; Vihman, 2017), research addressing their interaction with spoken language production has often been restricted to production accuracy or duration measures as metrics for assessing spoken language proficiency (e.g., Edwards et al., 2004; Munson et al., 2005). Likewise, speech motor control studies have provided in-depth analyses of developmental changes in articulatory variability, or movement velocity during word or sentence production (Smith and Goffman, 1998; Smith and Zelaznik, 2004; Green et al., 2010) without equivalently thorough assessments of children’s phonological or lexical knowledge allowing developmental interactions to be evaluated. Despite a certain imbalance in the focus and analytical approaches of interaction studies, the findings suggest that spoken language proficiency entails dynamical interactions among a set of language-related domains including speech motor skill.

In the present research, we adopted an integrated approach to the study of spoken language development considering parallel developments of the lexical, phonological, and speech motor systems. The study more specifically investigated interactions between domains that have not yet been empirically connected: in particular *phonological awareness*, the awareness of the particulate nature of the language (e.g., Fowler, 1991; Studdert-Kennedy, 1998, 2005) that develops with literacy (reviews in Anthony and Francis, 2005; Brady et al., 2011; Goswami and Bryant, 2016; in German: Fricke et al., 2016) and *anticipatory coarticulation*, a mechanism that is deeply rooted in kinematics (e.g., Parush et al., 1983) and motor planning (e.g., Whalen, 1990; Levelt and Wheeldon, 1994; Grimme et al., 2011; Perrier, 2012; Davis and Redford, 2019) and is fundamental to speech fluency.

While phonological awareness and coarticulatory mechanisms may in principle belong to different realms, we argue that they are developmentally strongly interconnected: phonological awareness relates to the ability to consciously extract functional units of phonological organization from the continuous speech flow (e.g., syllables, segments) and combine those discrete units into new sequences of variable size and meaning (e.g., Metsala, 2011). Coarticulation embodies speakers’ structural knowledge of the language, combining and (re)modeling its elementary particles into continuous articulatory movements and acoustic streams, hence contextualizing abstract representations into a decipherable “speech code” (Liberman et al., 1974; Fowler et al., 2016). In this perspective, investigating developmental changes in children’s coarticulatory processes may give us an opportunity to track how a combinatorial principle is situated within the representational and production levels and to capture more broadly how motor and cognitive functions come together to develop the skill of spoken language.

While children’s speech organization very early reflects their ability to combine phonetic units, the explicit awareness of the combinatorial nature of their native language forming larger compounds from smaller-sized units follows a more protracted development and seems to climax around the time children acquire literacy (e.g., Gillon, 2007). During that period, a gain in phonological awareness allows children to convert the already acquired phonetic units (i.e., sounds they hear and produce by means of distinct speech gestures) into phonological units. However, whether the acquisition of phonological knowledge only relates to attaining literacy or also modifies children’s spoken language organization in fundamental ways remains an empirical question. The alternative direction in which a gain in spoken language practice would stimulate the development of phonological awareness and literacy has also not yet been demonstrated. The present study provides a first step toward addressing this issue by testing whether lexical and phonological skills interact with speech motor control in development. More specifically, we examined whether children with greater knowledge of the segmental makeup of words in their native language exhibited a segmentally specified organization of their speech gestures and reflected in their coarticulatory patterns. We focused on the period encompassing kindergarten to the end of the first primary school year, which is relevant for phonological development as well as for attaining literacy. Our motivations driven from empirical research are further outlined below.

### What Are Children’s Units of Spoken Language Organization

In the last decades, a growing number of developmental studies in the area of spoken language ability have focused on *coarticulation degree,* which characterizes the extent to which the articulatory gestures for neighboring phonemes overlap temporally (e.g., Browman and Goldtstein, 1992). Looking specifically at *lingual* coarticulation, which regards the gestural organization of the tongue, some research has found a developmental decrease in vocalic anticipatory coarticulation over previous segments, within the syllables (e.g., Nittrouer et al., 1996; Zharkova et al., 2011; Noiray et al., 2018) and beyond the syllabic span (e.g., Nijland et al., 2002; Rubertus and Noiray, 2018). On the basis of these results, Noiray et al. (2019) reasoned that spoken language fluency may entail a gradual narrowing of speech units toward smaller-sized units. In young children, vowels may represent building blocks, which children organize their speech around because of their perceptual salience, long duration, and earlier acquisition compared to consonants (e.g., Polka and Werker, 1994; review Nazzi and Cutler, 2019). Hence, children’s vocalic and consonantal gestures may be activated more simultaneously than in adults, resulting in an overall larger vocalic influence on previous consonants and a greater degree of vocalic coarticulation than for adults. Instead, adults have been found to organize their speech with more temporally individuated gestures (Abakarova et al., 2018; Rubertus and Noiray, 2018). The result of rather large unit size speech organization echoes the multiple findings of whole-word learning (Vihman and Velleman, 1989; Keren-Portnoy et al., 2009; Menn and Vihman, 2011), transitional probability across syllables (e.g., Jusczyk et al., 1993; Saffran et al., 1996), or lexically grounded phonological development and production accuracy (Edwards et al., 2004; Velleman and Vihman, 2007; Vihman and Keren-Portnoy, 2013). The opposite finding of a lesser degree of coarticulation between consonants and vowel gestures in children compared to adults has also been reported (e.g., Katz et al., 1991), favoring a more segmental perspective of early spoken units.

Based on our own in-depth examinations of coarticulatory mechanism in both adults (Abakarova et al., 2018) and children (Noiray et al., 2018; Rubertus and Noiray, 2018), we have argued that (young) speakers exhibit gradients of coarticulation degree within a continuum from a more syllabic to a more segmental organization. The degree to which segment overlap depends on the gestural demands associated with the combined segments. In adults, contextual differences in coarticulation degree are well attested (e.g., Recasens, 1985; Fowler, 1994). For instance, syllables recruiting a single organ for the consecutive production of both consonantal and vowel targets (e.g. the tongue in /du/) require from speakers a functional differentiation between the subparts of the tongue (tongue tip, tongue dorsum). This type of syllable further requires greater spatiotemporal coordination in comparison to syllables recruiting two separate primary organs (e.g., the lips and tongue dorsum in /bi/). This phenomenon described within the theory of *coarticulatory resistance* has been reported in adults across languages over the past decades (review in Recasens, 2018). In children, extensive kinematic investigations of coarticulatory processes have been more challenging and hence somewhat restricted in scope compared to adults (e.g., limited variety of stimuli that can be tested in the young age, age range, sample size, scarcity of methodological replications across studies). Yet, once these studies are examined together, they support the view of coarticulatory gradients as observed in adults. While children show overall greater coarticulation degree than adults, they also exhibit contextual effects on coarticulation degree, which result from the particular combination of gestural goals between individual consonants and vowels. Based on those observations, we recently suggested a gestural approach as a “unifying organizational scheme to relate adults’ to children’s patterns. How coarticulatory organization matures over time is then no longer solely a question of direction (toward a greater or lesser coarticulatory degree) or categorical change in phonological organization (e.g., into segments or syllables) but a question of how a primitive gestural scheme shares similar tools (the articulators of speech), constraints, and principles (dynamic interarticulator coordination over time) with adults to instantiate complex phonetic combinations in line with the native language’s phonological grammar” (Noiray et al., 2019, p. 3037). In this context, the question of (early) units of speech production may be viewed as a part-whole interaction.

### The Development of the Lexical, Phonological, and Motor Domains

While the maturation of the speech motor system is central to spoken language fluency, lexical and phonological developments are equally crucial (e.g., Smith et al., 2010; Edwards et al., 2011), and research has suggested that they interact dynamically over time (e.g., Beckman et al., 2007; Sosa and Stoel-Gammon, 2012; Vihman, 2017). A main hypothesis motivating the present study is that adults’ coarticulatory patterns do not differ from those of children on the sole basis of greater precision of control from children’s speech production system. Adults also have (1) built an expressive lexicon from which to harness their phonological representations, (2) they have gained an explicit understanding of the structure of their language, and (3) an ability to manipulate this information into a quasi-infinite set of intelligible spoken forms. Hence, considering speech motor development as a goal-directed process – for example, speaking a language fluently – what distinguishes children from adults is that children have not yet built explicit correspondences between phonetic segments and their motor realizations. The rapid growth of the expressive lexicon observed during the kindergarten-to-school years may help children solve this correspondence problem and more generally develop stable relations between representational and executional levels. Vocabulary is indeed often considered the backbone of language acquisition, supporting the development of phonological representations (e.g., Ferguson and Farwell, 1975; Metsala, 1999) and production accuracy (e.g., Edwards et al., 2004; Nicholson et al., 2015). Previous research also suggests that children first develop articulatory “routines” for the syllables present in their expressive repertoire (e.g., Menn and Butterworth, 1983; Munson et al., 2005; Ziegler and Goswami, 2005; Vihman, 2017). This lexically based process may lay the ground for increased phonetic distinctions along the dimensions of height, fronting and rounding for vowels, place and manner of articulation for consonants, and the maturation of coarticulatory flexibility for a wider range of phonetic environments.

**Figure 1:**
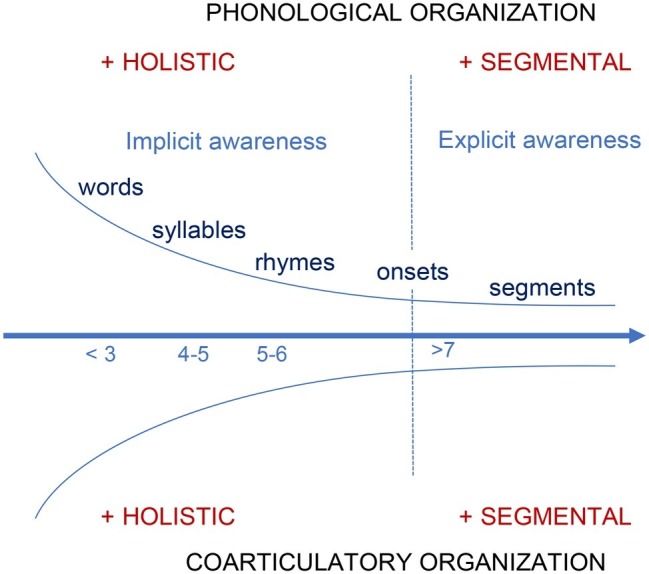
Theoretical conceptualization of the parallel development of phonological awareness and coarticulatory organization from holistic to more segmental organizations. The horizontal arrow (*x*-axis) illustrates developmental time (age in years). The curves indicate the nonlinear change in phonological and coarticulatory organizations over time.

This knowledge is at first experience-based; before entering primary school, children have limited *explicit* knowledge about the structural organization of their native language, that is, they have limited conscious awareness that the words they hear can be segmented into smaller-sized units (and recombined into new forms; e.g., Liberman et al., 1974; Gillon, 2007). Note that while the development of phonological awareness differs as a function of orthographic transparency (e.g. Fricke et al., 2016) or the age at which children learn how to read (e.g., review in Wimmer et al., 2000; Mann and Wimmer, 2002; Schaeffer et al., 2014; Goswami and Bryant, 2016) on average, children in kindergarten show only more or less equivalent proficiency in syllabic units’ awareness to that of school-aged children (in English: e.g., Liberman et al., 1974; in German: Ziegler and Goswami, 2005; Schaeffer et al., 2014) but no advanced phonemic awareness before explicitly learning how to read. Taken together, young listener-speakers would progressively access smaller units allowing them to decipher a wider range of speech forms and manipulate those flexible units to craft increasingly more complex speech flows. Figure 1 provides an illustrative conceptualization of these seemingly parallel developmental trajectories, from more holistic access and production of large units (e.g., lexemes) to more segmentally specified representations and coarticulatory organizations. Developmental overlaps (e.g., from lexeme access to rhyme access) and short-term regressions between learning phases may at times occur (e.g., Anthony et al., 2003), as noted in other domains (e.g., “phonological templates” during early word production: Vihman and Vihman, 2011; lip-jaw movement variability: Green et al., 2002; walking: Thelen and Smith, 1994). The developmental pace may also well change over time, as in other domains (e.g., speech motor control: Green et al., 2010). Figure 1 highlights the nonlinearity of those developmental processes over time (blue descending and ascending curves). With an advanced knowledge of their native language and a mature control of their speech motor system, adults naturally exhibit more flexible, context-specific organizations with greater or lesser coarticulation degree depending on the gestural properties of the individual segments assembled with one another.

Overall, results from these separate literatures suggest that the development of lexical, phonological, and speech motor abilities are fundamental to the maturation of children’s spoken language. However, to our knowledge, empirical studies examining their interactions with precision have been rare, and this gap has prevented a unifying account of spoken language development. The central hypothesis driving our current research is that the transition from the rather self-paced development of large unit phonological awareness to the more explicit knowledge of the phonemic constituents of the language initiated in primary school should correlate with a significant change in spoken language production from an experience-based holistic organization to a structurally informed, segmentally specified organization of spoken language. Because quantitative longitudinal investigations over a 2- to 3-year span are extremely difficult to conduct, we first opted for a cross-sectional examination of a sample of 41 children in the last 2 years of kindergarten (at 4.5 and 5.5 years of age) and the end of the first grade (at age 7). The latter cohort was chosen to ensure children have been exposed to explicit literacy instruction for a year. With this approach, we first tested for significant interactions between children’s motor, lexical, and phonological skills. Potential implications for causal relations are laid out in the discussion.

Based on our previous research, we expect differences in intra-syllabic coarticulation degree between children and adults but not necessarily between all child cohorts (Noiray et al., 2019). We also anticipated consonantal effects on children’s lingual coarticulatory patterns within each age cohort as found in a preceding study investigating children’s intra-syllabic coarticulation from the age of 3 (Noiray et al., 2018). More specifically, we expected a lower degree of lingual coproduction for consonant-vowel syllables requiring two constriction goals by spatially distinct articulatory organs than from those requiring two constriction goals by a single organ as found in adults (e.g., Iskarous et al., 2013; Abakarova et al., 2018), albeit to a lesser extent than adults. Importantly, expanding on previous research, we predicted greater phonological awareness and vocabulary would coincide with lower coarticulation degree, i.e., greater segmental differentiation of consonants and vowels in syllables. We further suspected interactions between motor and cognitive domains to be nonlinear and to reflect the complex dynamics in place during the development spoken language fluency. If this were found, it would suggest that the skill of spoken language fluency is not solely tied to production-related considerations but may instead result from and be an integral part of multiple interactions, which are fundamental to the development of each individual skill. If no correlation was to be found, it would on the contrary indicate that representational and production levels may not be tightly coupled in the sense that greater awareness of phonological discreteness does not interact with coarticulatory degree.

## Materials and Methods

### Participants

Forty-one monolingual German children all living in the Potsdam region (Brandenburg) were tested: ten 4-year olds (6 females, mean age: 4; 06, called K1 in subsequent analyses), thirteen 5-year-old children (7 females, mean: 5; 06, called K2 hereafter) in kindergarten, and eighteen 7-year-old children at the very end of the first or very beginning of the second grade in primary school (12 females, mean: 7; 02, called P1 hereafter). The discrepancy in sample size was due to greater difficulty in recruiting children in kindergarten. All children were raised in monolingual German families without any known history of hearing, language, or cognitive impairment. They were recruited *via* the child registry from the BabyLab of the University of Potsdam. Ethics approval was obtained from the Ethic Committee of the University of Potsdam prior to the study. All parents were also fully informed of the study and gave written consent for their child to participate.

### Production Task

The speech production task consisted in the repetition of trochaic pseudowords (i.e., conforming to German phonotactics) of the form consonant_1_-vowel-consonant_2_-schwa (**C**_**1**_**V**C_**2**_ǝ). Target phrases used as stimuli were pre-recorded by a native German female adult speaker. Three consonants varying in place of articulation: /b/, /d/, and /g/ and six tense, long vowels /i/, /y/, /u/, /a/, /e/, and /o/ were used. Pseudowords were chosen instead of real words to combine consonants and vowels varying in lingual gestures and coarticulatory resistance. Target pseudowords were embedded in a carrier phrase with the article /aɪnə/ resulting in utterances such as /aɪnə ba:də/. Utterances were repeated six times in semi-randomized blocks. To measure lingual coarticulation, we employed the technique of ultrasound imaging (Sonosite edge, fps: 48 Hz) that permits recording movement from participants’ tongue over time while producing various speech materials (Noiray et al., 2013). In this study, tongue imaging was integrated in a space journey narrative to stimulate children’s motivation to complete the task. Children were seated in a pilot seat with seatbelts, facing the operating console from a space rocket replica. The ultrasound probe on which children positioned their chin was integrated into a customized probe-holder as part of the rocket console (for a full description of the method, see Noiray et al., 2018). The acoustic speech signal was recorded synchronously with the ultrasound tongue video *via* a microphone (Shure, fps: 48KHz).

### Assessment of Phonological Awareness and Vocabulary

Assessments of various levels of phonological awareness (rhyme, onset segment, and individual phonemes) were conducted with the Test für Phonologische Bewusstheitsfähigkeiten (TPB; Fricke and Schäfer, 2008). Prior to testing, children were familiarized with all images used as test items. The procedure for each of the TPB test is briefly summarized below; a complete description can be found by Fricke and Schäfer (2008). The tests were scored according to the test instructions, and raw scores were considered for subsequent analyses.

#### Rhyme Production

Children are shown a picture and are instructed to produce (non)words that rhyme with the word corresponding to the target picture (e.g., *Puppe: Muppe, Kuppe, Wuppe*). Children are instructed to provide as many rhymes as they can. However, to make the task comparable for every child, we scored children’s proficiency differently from the test instructions: for each of the 12 target words, children scored 1 point if they succeeded in giving at least one correct rhyme; if not, they scored zero. This way, we could assess the stability and generalization of the rhyming skill rather than relying on raw number of rhymes produced (e.g. if a child produced six rhymes for two target words but then failed for all other target words).

#### Onset Segment Deletion

Children are shown a picture and are instructed to delete the onset segment from the word represented by the picture and utter the resulting nonword (e.g. Mond: ond; Zahn: ahn). Note children were precisely instructed what to delete (e.g. “delete “m” from Mond”). A total of 12 words is tested in each age cohort.

#### Phoneme Synthesis

Children are instructed to produce a word after hearing a pre-recorded female voice uttering its phonemes one by one (e.g. fee: [f-e:], dose: [d-o:-z-Ə], salat: [z-a-l-a:-t]). For the onset segment deletion task, the TPB assessment uses a total of 12 words for each age cohort.

#### Expressive Vocabulary

Expressive vocabulary was tested with Patholinguistische Diagnostik bei Sprachentwicklungsstörungen (PDSS; Siegmüller and Kauschke, 2010) and widely used to assess German children’s lexical repertoire. The test consists of a 20-word picture naming task assessing nouns for the target ages (see Table 1 for an overview). In subsequent analyses, we used a composite score for phonemic awareness (PA hereafter that includes the two tasks tapping phoneme-size awareness: onset deletion and phoneme synthesis).

**Table 1:** Summary of the results from the assessments tapping phonological awareness (Rhyme, Composite PA) and expressive vocabulary (VOC) conducted in 4-year-old (K1), 5-year-old (K2), and 7-year-old children at the end of first grade (P1).

Descriptive statistics for phonological awareness and vocabulary assessments				
	Task	Cohort	Mean score	Range
**Phonological awareness**	Rhyme production	K1	8.09	0–12
		K2	9.15	8–12
		P1	11.33	4–12
	Composite PA	K1	0	0
		K2	1.00	0–13
		P1	19.6	11–24
**Lexicon**	VOC	K1	26.1	22–30
		K2	27.38	24–32
		P1	32.11	30–36

We focused on *output* phonological tasks as well as *expressive* vocabulary because we were interested in their direct relationship with children’s speech production. Given that young children have a limited attention span, we could also assess children’s actual proficiency with better confidence than when conducting long series of cognitively demanding assessments. All assessments were conducted in our laboratories by experimenters trained by a speech language pathologist.

## Statistical Analyses

Consistent with previous research, intra-syllabic coarticulation degree was estimated in terms of whether the lingual gesture for a target vowel was anticipated in the previous consonant (see review on vowels’ degrees of aggressiveness in the context of different consonants: Iskarous et al., 2010). We focused on the antero-posterior tongue dorsum position that is highly relevant in terms of articulatory and acoustical contrasts between vowels (e.g., Delattre, 1951). We calculated differences in tongue dorsum position between the production of consonants and following vowels. A tongue dorsum position for a consonant (e.g., /g/) that varies in the context of various vowels (e.g., /a/, /i/) indicates vocalic anticipation onto the previous consonant and hence a high coarticulation degree. On the contrary, low coarticulation degree is reflected by an absence of change in tongue dorsum position during the consonant in the context of various vowels (review in Iskarous et al., 2010).

Differences in coarticulation degree were estimated for each consonantal context from the midpoint of the consonant (C_1_) compared to the vowel midpoint (V). A few preliminary processing steps were necessary. First, the corresponding midsagittal tongue contours for both C_1_ and V were extracted from the ultrasound video based on the acoustic speech signal labeling. The tongue contours were then analyzed using SOLLAR (Noiray et al., submitted), a platform created in our laboratory for the analysis of kinematic data (Matlab environment). For each target tongue contour, a 100-point spline was automatically generated, and the *x*- and *y*-coordinates for each point were extracted. In subsequent analyses, we used the horizontal *x*-coordinate for the highest *y*-coordinate point of the tongue dorsum to reflect its variation in the anterior-posterior dimension (e.g., anterior position for /i/, posterior position for /u/, e.g., Abakarova et al., 2018). Data were normalized for each participant by setting the most anterior tongue dorsum position during the target vowel midpoints to 0 and the most posterior tongue dorsum position to 1. Tongue dorsum positions for consonant midpoints were then scaled within this range.

To test for developmental differences in coarticulation degree, we employed linear mixed effects models (LMER), using the “lme4” package in R (version 1.1–19; Bates et al., 2015). Coarticulation degree was calculated by regressing the horizontal position of the tongue dorsum at consonant midpoint (PEAKC_1__X) on the horizontal position of the tongue dorsum at vowel midpoint (PEAKV_X) for each age group (K1, K2, and P1). Two interaction terms were used: Coarticulation and Consonant (C_1_) and Coarticulation and Age. By-subject C_1_ and by-word random slopes for PEAKV_X were included as random effects.

To test for an effect of phonological awareness and vocabulary on children’s coarticulation degree, we then employed *Generalized Additive Modeling (GAM),* a statistical approach allowing us to test for linear and nonlinear relationships (Winter and Wieling, 2016; Wood, 2017; for a comprehensive tutorial, see Wieling, 2018). To date, this approach has only been used in psycholinguistic research with adults (e.g., Strycharczuk and Scobbie, 2017; Wieling et al., 2017) and only recently in the developmental domain (Noiray et al., 2019). In this study, we were interested in the effect of three variables on the degree of coarticulation: RHYME, COMPOSITE_PA (a composite computed from the sum of the scores obtained for both phonemic awareness tasks: onset segment deletion and phoneme synthesis, see section “Descriptive Statistics for Phonological Awareness and Vocabulary”), and VOC. We used the function *bam* of the *mgcv* R package (version 1.8–26) and *itsadug* (version 2.3). Our dependent variable was again PEAKC_1__X with respect to PEAKV_X. We predicted this value on the basis of a nonlinear interaction, which is modeled by a tensor product smooth (te). A tensor product smooth can model both linear and nonlinear effects across a set of predictors and their interaction (see Wieling, 2018) here between: RHYME, COMPOSITE_PA or VOC, and PEAKV_X. The resulting estimated degrees of freedom (edf) indicate whether the relation is linear (value close to 1) or nonlinear (values above 1).

## Results

### Testing for Developmental Differences in Coarticulation Organization

Table 2 shows the results from the LMER testing for age-related differences in coarticulation degree across all consonants and vowels. No significant difference was noted across the three target age groups. However, differences in coarticulation degree were found across consonantal contexts, with a lower coarticulation degree in alveolar /d/ context as compared to labial /b/ context (estimate: −0.11793, *p* < 0.05). Coarticulation degree did not differ across other consonantal contexts.

**Table 2:** Results from the linear mixed effects model testing for age comparisons in coarticulation degree between the 4-year-old group (K1), 5-year-old group (K2), and 7-year-old group (P1).

Age comparisons	Estimate	Std. error	*t*	*p*
K2–K1	0.016	0.057	0.3	0.765
P1–K1	−0.061	0.05	−1.225	0.228
P1–K2	0.077	0.046	−1.67	0.102

### Descriptive Statistics for Phonological Awareness and Vocabulary

Pearson product-moment correlations were computed to assess relationships between all developmental assessments. For the rhyming task, we conducted the task in 40 of the 41 children because one P1 child did not want to conduct the rhyming task. A strong positive 0.94 correlation (*p* < 0.001) was found between scores for onset deletion and phoneme synthesis. In subsequent analyses, testing the effect of phonological awareness on coarticulatory organization, we therefore computed a composite score as the sum of the scores obtained in the two tasks. This score was taken to reflect children’s phonemic awareness (COMPOSITE_PA), that is, of phonemic units in comparison to the awareness of larger phonological units (rhymes).

**Figure 2:**
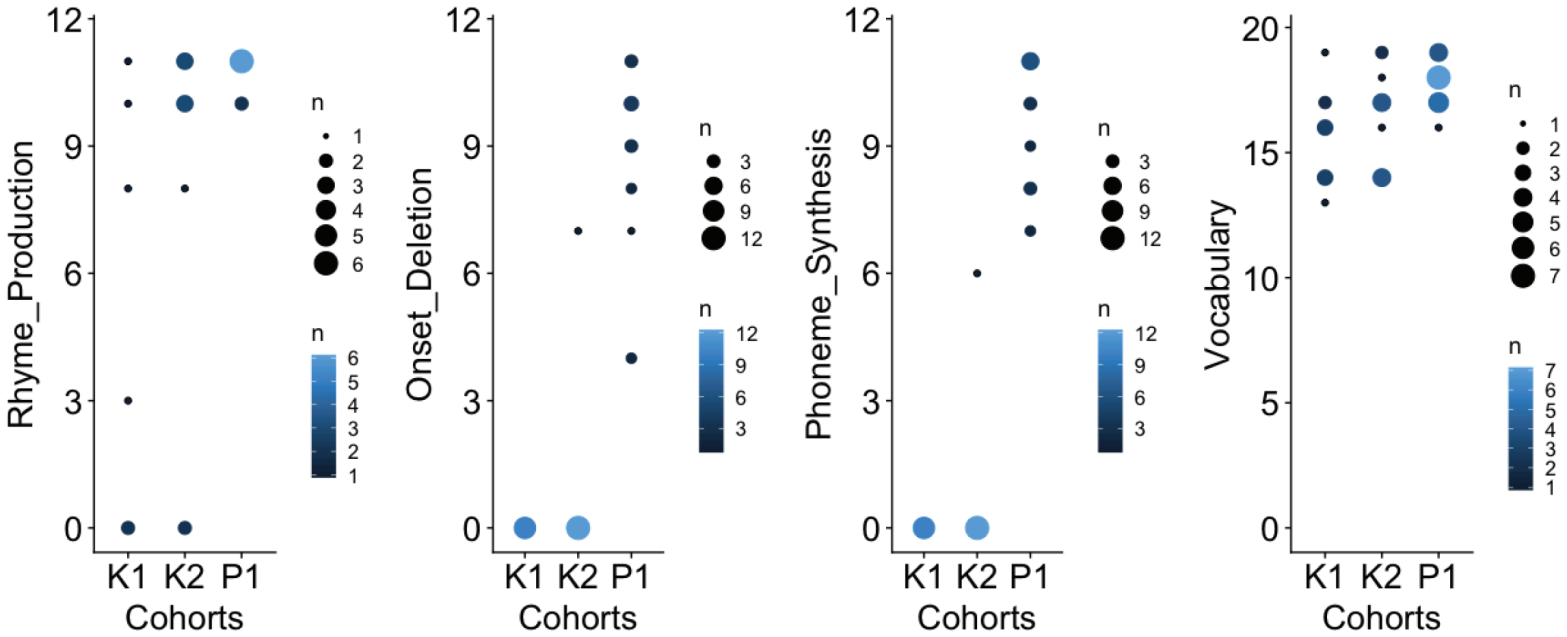
Score distribution for each of the four developmental assessments conducted across age groups (K1, K2, and P1). From left to right: rhyme production, onset deletion, phoneme synthesis, and vocabulary. The filled colored circles from different sizes represent different numbers of participants sharing a similar score.

Figure 2 provides an overview of the score distribution for each of the four developmental assessments conducted across child cohorts. Dot plots were used to highlight variations in the number of children obtaining a target score. Table 1 provides a summary of the descriptive statistics reflecting children’s phonological awareness and expressive vocabulary. Mean score and range reflect the number of correct items (raw scores). While mean scores increased with age for all language-related skills, results (1) revealed stark individual differences within the same age-group and (2) overlap in scores across age groups for rhyme and expressive vocabulary. For the phonological tasks targeting the awareness of phonemic units (onset segments and individual phonemes), children in kindergarten had overall great difficulty completing the tasks (despite being familiarized with pre-test items), while children in the first grade could complete the tasks with various levels of proficiency.

The Welch *t* test was conducted to test for developmental differences in phonological awareness and vocabulary. Performance on rhyme production for the scoring procedure we employed did not yield any significant differences among age groups (K1–K2: *t* = −0.58, df = 17.47, *p* < 0.6; K1–P1: *t* = −0.58238, df = 17.47, *p* < 0.6; K2–P1: *t* = −1.9085, df = 12.524, *p* < 0.08). With regard to the composite score computed to target the awareness of phonemic units, 5-year-old children (K2) did not differ in performance from 4-year olds (K1) (*t* = −1, df = 12, *p* < 0.4). Only 7-year-old children (P1) showed greater proficiency than K2 (*t* = −15.572, df = 21.128, *p* < 0.0001 4.693e-13) and K1 (*t* = −30.006, df = 14, *p* < 0.0001). Hence, a developmental increase in awareness of segmental units was found between children in kindergarten altogether and those in the first year of primary school, which yielded an overall high correlation between age and PA composite of 0.9 (*p* < 0.0001). Regarding vocabulary, similar directions were found. K1 children did not exhibit lower proficiency than K2 (*t* = −0.95914, df = 19.728, *p* < 0.4), only when compared to P1 children (*t* = −7.0665, df = 16.375, *p* < 0.0001). K2 children also had lower vocabulary scores than P1 children (*t* = −4.0338, df = 16.257, *p* < 0.001). However, unlike for phonemic awareness, the correlation between age and vocabulary was not significant (0.12, *p* < 0.3).

### Interaction Between Phonological Awareness and Coarticulation Degree

Given the results from the developmental assessments, we adopted the following statistical approach: we first tested the interaction between *rhyme* proficiency as an index of intermediate unit-size awareness and coarticulation degree for all children. We then further tested for a separate interaction between phonemic awareness (COMPOSITE_PA, named PA for short hereafter) or vocabulary (VOC) and coarticulation degree. We conducted GAM analyses to illuminate potentially nonlinear interactions.

First and foremost, an interaction between rhyme awareness and coarticulation degree was found across all three consonantal contexts (*p* < 0.0001). More specifically, greater rhyming skills were associated with lower coarticulation degree. Furthermore, the estimated degrees of freedom (edf) were all above 1, which indicates that rhyme proficiency was non-linearly related to an increase in children’s coarticulation scores. Nonlinear interactions between rhyme and coarticulation degree were found in each consonantal context (Table 3). The nonlinearity was the highest in the alveolar context (edf: 10.778), followed by the velar and labial contexts. This means that the pattern of interaction between rhyme and coarticulation degree was specific to the gestural organization of the consonant-vowel combinations.

**Table 3:** Tensor smooth terms of the generalized additive model testing for an interaction between rhyme and coarticulation degree for all children per consonantal context /b/, /d/, /g/. edf: estimated degrees of freedom.

Tensor smooth functions (te)	edf	*f*	*p*
te(Rhyme): consonant /b/	4.552	211.43	<0.0001
te(Rhyme): consonant /d/	10.778	24.76	<0.0001
te(Rhyme): consonant /g/	9.583	42.02	<0.0001

**Figure 3:**
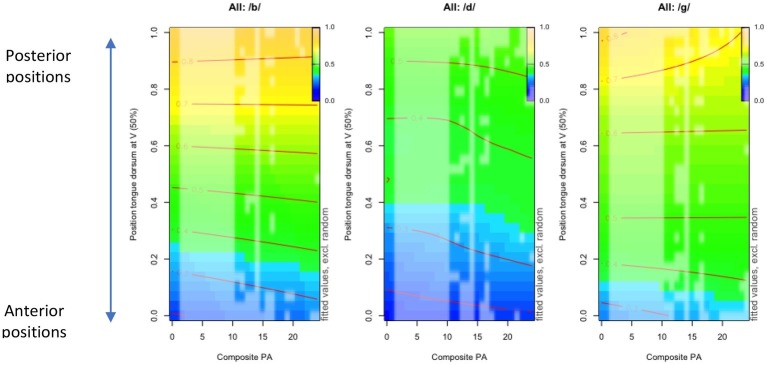
Terrain maps illustrating changes in the tongue dorsum gesture across three consonantal contexts (/b/: left column, /d/: middle column, /g/: right column) as a function of tongue dorsum position for target vowels (*y*-axis) and composite phonological awareness scores from 0 (the minimal score obtained) to the maximal score of 25 (*x*-axis).

Table 4 presents an overview of the GAM model testing for an interaction between phonemic awareness (PA) and coarticulation degree. A negative correlation was found, that is, greater phonemic proficiency coincided with lower coarticulation degree. This interaction differed significantly across consonant contexts (*p* < 0.0001). The nonlinearity of the interaction was again the most prominent in the alveolar context and lowest in the labial context. Figure 3 presents three-dimensional visualizations of the nonlinear interaction patterns obtained for each consonantal context, called terrain maps. These visualizations (also called contour plots) provide further insights into the direction of the observed interaction between PA and coarticulation degree. More specifically, they depict differences in the tongue dorsum position during the production of each stop consonant (/b, d, g/ from left to right plot) with respect to the tongue dorsum position during the production of the subsequent target vowel (*y*-axis) as a function of children’s PA score (*x*-axis). In the plot, changes are expressed by means of a color scaling. The color scheme in the small upper right rectangle provides a referential color coding for various tongue dorsum positions scaled from 0 to 1. While blue shades characterize more anterior tongue dorsum positions (as expected for anterior vowels such as /i/), orange shades correspond to more posterior tongue positions (e.g., for /u/). The full-size plots themselves display the tongue position during the consonant as a function of its subsequent vowel position (*y*-axis) and PA scores obtained (value on the *x*-axis). If the tongue dorsum position of the consonant is highly influenced by the upcoming vowel (i.e., if coarticulation degree is high), the color distribution within the plots is expected to resemble the referential color scaling provided for the vowel tongue dorsum positions (i.e., yellow color for more posterior and blue color for more anterior tongue dorsum positions). The red contour lines are used similarly to isolines in topographic maps (e.g. for hiking) to indicate locations sharing the same (predicted, based on all trials) value. Here, the values are not altitude landmarks, but tongue dorsum positions. Hence, red contour lines characterize locations of identical consonant tongue dorsum positions across a set of PA scores (from 0 to 24) as a function of their vocalic environment. The direction and shape of the contour line provide information whether changes in tongue dorsum position are linear (straight line) or not (curved line).

**Table 4:** Tensor smooth terms of the generalized additive model testing for an interaction between phonemic awareness (composite_PA) and coarticulation degree for all children per consonantal context /b/, /d/, /g/.

Tensor smooth functions (te)	edf	*f*	*p*
te(composite_PA): consonant /b/	3.000	411.73	<0.0001
te(composite_PA): consonant /d/	13.879	17.73	<0.0001
te(composite_PA): consonant /g/	8.049	52.94	<0.0001

Let us now take a concrete example. In the labial context /b/, we can see that for a target vocalic tongue dorsum position of 0.3 (value on the y-axis), the corresponding position at the consonant midpoint is about 0.4 (value on the red contour line) for children who have obtained a PA score close to 0. From a score of 10 upward, the tongue dorsum position during the consonant becomes slightly more posterior (i.e., above the 0.4 red contour line, hence further away from the target 0.3 value for its subsequent vowel).

Moving on to the alveolar context, it can be noted that the position of the tongue dorsum during the alveolar /d/ stop remains overall in a central (green shade) to anterior position (blue shade) regardless of the upcoming vowel. This shows that the tongue dorsum position during the alveolar stop resists vocalic influences due to more immediate gestural constraints requiring a more anterior to central tongue dorsum position. However, scores starting from 10 (about half the maximal score) onward are associated with a change toward a more central tongue dorsum position as compared to children with poorer PA scores. In labial and velar contexts, the color scaling characterizes more faithfully the range of vocalic targets in the antero-posterior dimension: from blue for anterior vowels to orange for more posterior vowels. This is very clear for children with a poor PA score: the tongue dorsum position for all vowels is well anticipated in the consonant. The color patterning differs in children with higher PA scores reflecting a more central tongue dorsum position (larger green portion) and hence a lower coarticulation degree. Furthermore, in velar context, the contour lines are flatter with central vowels (e.g., on *y*-axis: 0.5–0.6 values) and more non-linear in the context of posterior vowels (0.8 and above). In the labial context, the interaction between phonemic awareness and coarticulation degree is slightly nonlinear (edf value: 3). In Figure 3, the red contour lines look overall flat, except with anterior vowels (e.g., 0.3 value and below). Overall, Figure 3 shows that the interaction of PA and coarticulation degree: (1) approximates linearity in labial and velar contexts contrary to the alveolar context and (2) varies as a function of the various combination of individual consonants and vowels. The implications of these nonlinear relationships between phonological and motor domains are discussed in section “Nonlinear Interactions Between Vocabulary, Phonological Awareness, and Coarticulatory Organization.”

These visual outputs differ markedly from standard numerical reports. They are quite valuable for speech production research in general and more so for the developmental field (e.g., Figure 3) because the continuous color scaling used in these plots can reveal gradients in target effects or interactions between parameters and hence potentially identifying nonlinear patterning. In the case of spoken language acquisition, these permit departing from categorization of children’s articulations in terms of abstract phonological targets (which they are in the process of acquiring) and instead obtain more faithful descriptions of the variety of articulatory expressions for a given target. This type of description is particularly relevant in the developmental field because like adults – and even to a greater extent than adults – children do not produce words or segments uniformly across repetitions. Acoustic and articulatory variability are indeed ubiquitous in child speech (e.g., Heisler et al., 2010). The color scaling in the GAM contour plots hence provides a fair depiction of the variations in tongue dorsum positions within regions associated with a specific target (e.g., individual vowels) or in interaction with a phonetic environment (e.g., a specific vowel in the context of a specific consonant).

### Interaction Between Expressive Vocabulary and Coarticulation Degree

**Figure 4:**
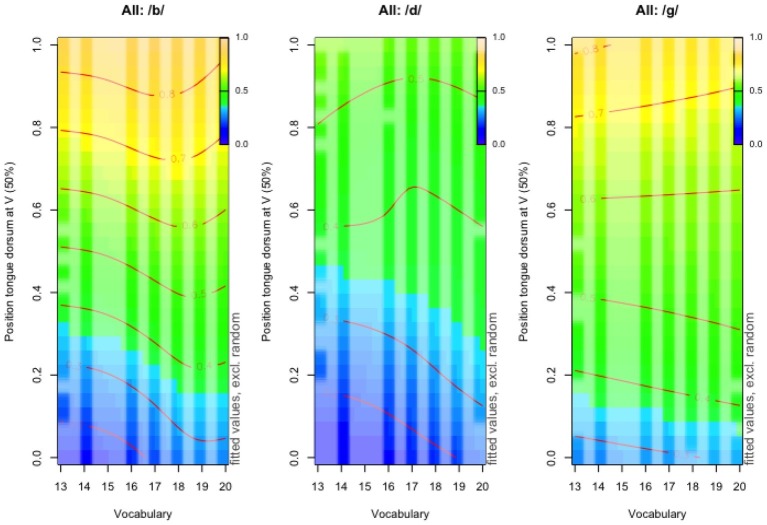
Terrain maps illustrating changes in the tongue dorsum gesture across three consonantal contexts (/b/: left column, /d/: middle column, /g/: right column) as a function of tongue dorsum position for target vowels (*y*-axis) and vocabulary scores from 13 (the minimal score obtained) to the maximal score of 25 (*x*-axis).

Last, we tested for an interaction between children’s expressive vocabulary and their pattern of coarticulation degree. A significant effect was found in all three consonantal contexts (Table 5, *p* < 0.0001). Overall, nonlinear patterns of interactions between domains were noted. However, those were not uniform across consonant and vowel combinations (Figure 4). In the labial context, an increase in vocabulary score coincides with lower coarticulation degree. For example, in anterior vowels that have a 0.2 tongue dorsum position value (*y*-axis), the corresponding tongue dorsum position during the labial stop production has a value of 0.3 in children with low vocabulary while close to 0.4 in children with advanced vocabulary. Similar trends are observed in syllables including an alveolar onset, but the interaction between vocabulary and coarticulation degree is this time more nonlinear (more pronounced curved lines) and complex than in the labial context. For children with more proficient vocabulary (e.g., score 16 upward), the tongue dorsum position is slightly more central in the case of anterior vowels (e.g., 0.2). Consonantal tongue positions in the context of central vowels (e.g., 0.6) are characterized by a slightly oscillatory behavior from more to less to more central. Last, tongue position for the alveolar stop flanked by posterior vowels (e.g., 0.8) also shows a nonlinear pattern with an overall central tongue dorsum position. Last, in the velar context, the relation between vocabulary and coarticulation degree also translates into slightly more central tongue dorsum positions in children with higher vocabulary score. To summarize, greater expressive vocabulary is associated with a more central tongue dorsum during the consonant and hence lesser influence from individual vowels.

**Table 5:** Tensor function terms of the generalized additive model testing for an interaction between expressive vocabulary and coarticulation degree for all children per consonantal context /b/, /d/, /g/.

Tensor functions (te)	edf	*f*	*p*
te(Vocabulary): consonant /b/	6.366	171.63	<0.0001
te(Vocabulary): consonant /d/	10.29	23.61	<0.0001
te(Vocabulary): consonant /g/	6.873	64.4	<0.0001

## Discussion

In this study, we asked whether children’s phonological awareness and expressive vocabulary have an impact on *anticipatory coarticulation*. Our general motivation for this research stemmed from independent findings made in speech motor control and developmental phonology suggesting an increasing access to and use of phonemic units during the kindergarten-to-primary school period. Results drawn from a cross-sectional investigation of 41 children provide the first empirical evidence that vocabulary and phonological awareness interact dynamically with coarticulation degree during the period from kindergarten to primary school. In general, greater phonemic awareness and vocabulary were associated with greater segmental differentiation of tongue gestures in children’s coarticulatory organization. We expand below on the implications of those findings for the development of spoken language fluency.

### Age-Related Versus Skill-Based Descriptions of Spoken Language Development

In the past decade, a fair amount of empirical research has reported greater vocabulary and phonological awareness in school-aged children than children in kindergarten (in German: Kauschke, 2000; Wimmer and Mayringer, 2002; Schäfer et al., 2014; in English: Carroll et al., 2003; Ziegler and Goswami, 2005). However, results from the present study suggest that age-driven categorizations are not always the only suitable ways to characterize skill development or at least they may underestimate its complexity. Several findings uphold this argument.

First of all, the language-related assessments conducted in this study provide a mixed validation of prior findings regarding a developmental increase in expressive vocabulary and phonological awareness. Indeed, our sample of kindergarten children was seemingly as proficient as first-grade children in expressive vocabulary as attested by the absence of significant age differences. Likewise, they were as proficient as first-grade children in their rhyming skills, which suggest that by the age of 4.5, they have gained awareness of *intermediate* size phonological components. This may be due to rhyming practices being initiated early in age, *via* singing, counting rhyming games at home or in kindergarten. With respect to tasks probing phonemic units, the two youngest cohorts did not differ from each other but showed significantly lower awareness than school-aged children at age 7. Interestingly in our study, the only 5-year old who could actually perform the phonemic task was able to read a few words and had knowledge about some letters. Hence, success in these tasks may emerge only once children have been explicitly trained in phonemic decoding/encoding, either in primary school in the context of reading acquisition (e.g., Ziegler and Goswami, 2005; Schaeffer et al., 2014) or with parents at home. We discuss this point further in section “An Integrated-Interactive Approach to Skill Development.”

Second, children within the same age group did not behave all in the same way but instead exhibited substantial individual variability (Figure 2), a phenomenon also previously noted (e.g., review in Sosa and Stoel-Gammon, 2012; see also Wimmer and Mayringer, 2002; Schäfer et al., 2014). In the present study, this was the case in all three age groups and for all assessments, except for tasks probing phonemic awareness in kindergarteners (onset segments, phoneme synthesis) for which we noted a floor effect. Regarding first-grade children, it seems that while they have gained substantial awareness of sub-lexical units in comparison to children in kindergarten, it takes longer to be fully proficient in manipulating phonemic units (cf. the scores distribution, Figure 2). Regarding vocabulary, wide disparities across children from the same age are well-established (e.g., CDI reports within and across languages). Similar conclusions have been drawn regarding children’s coarticulatory patterns (e.g., at 4 years of age in Nittrouer and Burton, 2005; Barbier et al., 2015; at 5 years of age in Zharkova, 2017; overlap between 3–4-year and 5-year olds in Noiray et al., 2019) and here again with no systematic age-related difference in coarticulatory degree across consonantal contexts.

It is not uncommon for developmental researchers to point to between-age overlaps and/or substantial within age-group differences in various abilities. The question is then why those differences are observed. A simple answer may be that children are at different individual stages in their developmental trajectory. For instance, well-attested vocabulary spurts seem to depend on pre-existing achievements (e.g. reaching the 50 words milestone) rather than be the result of biological age progression (see review of lexical development in Nazzi and Bertoncini, 2003). Other studies have underlined stronger developmental dependencies based on proficiency rather than age (e.g., between phonological development and motor ability, e.g., Smith, 2006; Goffman, 2010; between vocabulary and production accuracy, e.g., Edwards et al., 2004; Vihman and Croft, 2007). When that is the case, age-related interpretations are problematic because they may attribute evidence (e.g., a decrease in coarticulation degree) to the wrong source or hide complex relationships between factors that are individual-specific rather than age-dependent. This is not to argue that age does not matter: the development of speech motor skill along with lexical and phonological knowledge can actually be described within a maturational perspective because all skills develop in the time domain. It is hence not surprising that correlations between age and phonological awareness were found in our study – albeit not with all PA tasks and not with vocabulary. However, while age-based descriptions of language acquisition may be interpreted in the perspective of biologically-driven developments, it may instead be the effect of experience upon the learning mechanism (i.e., the exposure to and practice speaking the language) that gives maturation its transformational power (e.g., in perception: Kuhl et al., 1992; Hay, 2018). Uncovering how experience shapes (spoken) language acquisition independent of age has been not only thrilling but also enduring challenge for psycholinguists because experience unfolds within an extended time scale and results from multiple interactions in a continuously variable environment that remains difficult to replicate in lab environments.

To summarize, the results reported in this study provide good incentives for future research to draw skill-based comparisons of children’s linguistic ability. With this approach, we will not only account for the complex developmental relationships across domains taking place in the first decade of life, we will also better capture the complexity of (spoken) language acquisition arising from both experience-based and biologically driven processes than if our analyses are restricted to age comparisons. This leads us to the discussion of the role of skill interactions for (spoken) language development.

### Nonlinear Interactions Between Vocabulary, Phonological Awareness, and Coarticulatory Organization

As reported in previous sections, no uniformly strong differences in coarticulation degree emerged between 4-, 5- and 7-year-old children (Table 2). However, children showing poor phonological awareness indicated overall greater coarticulation degree than children with higher scores. This suggests that for children with poorer phonemic representations, lingual gestures for consecutive consonants and vowels may be activated together with substantial vocalic anticipation. Further, we noted no uniform relation between coarticulation and phonemic awareness across children’s scores, by which each unit change in one domain would result in an equivalent (linear) unit change in the other domain of interest. In our sample of children, the relationship between domains was non-linear and therefore more complex: an increase in children’s phonemic awareness score was at times not associated with any equivalent change in coarticulatory pattern until reaching a certain stage. Last, those non-linear interactions varied across phonetic contexts (cf. edf values). The shape of the skill interactions indeed differed as a function of the identity of the coarticulated consonants and vowels and the compatibility of their gestural goals (cf. colored terrain maps). For instance, in the case of a syllable involving two gestures from two anatomically distinct organs (the lips for the labial /b/ and the tongue for any vowel), vocalic influences remained high regardless of children’s phonemic proficiency (rather flat isolines and all colors well represented; Figure 3). However, in the context of the alveolar /d/ stop that involves two consecutive lingual gestures within a short-temporal span (tongue dorsum for both /d/ and subsequent vowels), non-linear interactions were more noticeable. Children with advanced awareness of the smallest phonemic units (e.g., higher scores) exhibited slightly more central tongue dorsum positions than children with poorer ability (larger blue portion characterizing an anterior tongue position). This suggests a gradual functional decoupling between the anterior (tip-blade) and the posterior subparts of the tongue (dorsum-back). While the tongue remains in a rather anterior position during the alveolar stop production, the tongue dorsum seems a little more central as if to anticipate the production upcoming vocalic gesture. Non-linear interactions were also visible in syllables including a velar onset. Variation in phonemic awareness coincided with variation in the palatal-to-velar constriction location as a function of the vowel (see Recasens, 2014). While lower phonemic awareness was associated with greater vocalic influences (full color scale represented, Figure 3), greater awareness correlated with more central tongue positions during the consonant articulation. This finding corroborates previous research reporting a lack of speech motor independence in the early age (e.g., Nittrouer et al., 1996) and provides additional evidence for an important interaction with phonemic awareness, which seems particularly relevant for the coarticulation of complex gestural goals involving a single organ.

Nonlinearities were also observed in the interaction between vocabulary and coarticulatory patterns. First, results indicated that children with greater expressive vocabulary showed lower intra-syllabic coarticulation degree independently of age (cf. 0.12 correlation) and hence greater sensitivity to the gestural demands underlying various consonant-vowel combinations, while children with poorer vocabulary showed larger coarticulatory units with greater vocalic influence over previous consonants. Given numerous findings supporting a lexically grounded development of phonological representations and its impact on production accuracy (e.g., Ferguson and Farwell, 1975; Metsala, 1999; Beckman and Edwards, 2000; Edwards et al., 2004, 2011; Munson et al., 2005; Vihman and Keren-Portnoy, 2013), our results supplement existing evidence that a rich lexical repertoire leads to greater phonological differentiation, by showing it may also support greater motor differentiation and flexibility in coarticulatory patterns depending on the gestural demands associated with consecutive segments. In the present study, the interaction between vocabulary and coarticulation degree in the alveolar context provides a compelling example that children with more proficient vocabulary show greater differentiation between the tongue dorsum and tongue tip for coarticulating consecutive consonantal and vocalic gestures recruiting the same organ. Second, the nonlinear nature of the interaction between vocabulary and coarticulation also suggests that the coupling between domains does not develop incrementally but rather that it may be when individual children reach a certain size of expressive vocabulary that the interaction with production weighs in children’s coarticulatory organization.

Taken together, results support the view of a by-stage approach to skill development. Milestones and developmental stages have long been identified in various developmental domains (e.g., walking: Thelen and Smith, 1994; perception: e.g. Best, 1994; Maye et al., 2002; Werker, 2018; spoken language: e.g., Kuhl, 2011; language processing: e.g., Vilain et al., 2019) and provide researchers with referential landmarks for a better understanding of typical trajectories, as well as useful tools for the diagnosis and prediction of potential deviations from typical pathways. In the domain of spoken language development, canonical babbling stands as an undisputed milestone allowing children to move toward a more complex quality of the speech production skill (e.g., production of the first meaningful words). This study points to a similar mechanism for skill interaction. In the same way children continuously develop individual skills (e.g., spoken language, expressive vocabulary), there may be milestones and developmental stages characterizing periods for which an interaction is (more significantly) activated. The outcome of this interaction would lead children to progress toward a new developmental stage. Taking again the relation between phonemic awareness and coarticulation, an average score reaching above 10 may characterize a developmental stage by which phonemic differentiation is maturing both at the representational and speech motor levels.

### An Integrated-Interactive Approach to Skill Development

In a preceding study, we had argued that the question “whether children organize their speech in segments versus syllables versus phonological words or lexical items is twofold: It requires finding the phonological units guiding children’s speech production and the motor units embedding those higher-level units” (Noiray et al., 2018, p. 8). The research conducted since then motivates us to endorse an integrated-interactive approach to (spoken) language acquisition. By integrated, we mean that the gradually acquired knowledge about different unit types and sizes does not constrain children to move from one organizational scheme to another (e.g., from holistic to segmental representation of speech or vice versa). Instead, this knowledge would integrate into an increasingly more complex and flexible language system allowing children to gradually manipulate a greater variety of phonetic compounds and structural organizations (Noiray et al., 2019). At the production level, this integrative process is exemplified in preschool-age children using gradients of coarticulation degree to accommodate the varying gestural demands of consecutive consonants and vowels (Noiray et al., 2019). At the representational level, the way phonological awareness has been traditionally assessed directly reflects an integrative approach to phonological development: children’s structural knowledge of their native language is usually tested incrementally with tasks tapping different levels of unit complexity (e.g., words, syllables, rhymes, and segments). Phonological awareness may therefore be envisioned as an integrative learning process: it is only once children have fully integrated all organizational levels and can manipulate them into various ways that they have reached adult-like phonological representations.

The process of combinatoriality is not unique to language. In their discussion of language discreteness, Studdert-Kennedy and Goldstein (2003) had remarked on striking structural similarities between the way languages pattern and the way other processes in nature pattern (e.g., in biology, physics, chemistry). They argue for a “particulate principle” (Abler, 1989) under which “units that combine into a larger unit do not disappear or lose their integrity: they can re-emerge or be recovered through mechanisms of physical, chemical, or genetic interaction, or, for language, through the mechanisms of human speech perception and language understanding” (Studdert-Kennedy and Goldstein, 2003, pp. 52–53). Congruent with this theoretical position, we consider a view of (spoken) language in which various structural types of combinations – gestures, segments, syllables, and words – are not mutually exclusive but reflect complementary levels of linguistic organizations that all contribute to the richness and complexity of language systems (e.g., Goffman et al., 2008; Noiray et al., 2019). From very early in development, the process of coarticulation itself binds gestures, sounds, phonetic units together to create compounds that ultimately lend meaning to speech streams. This imparts to coarticulation a special role for (spoken) language development beyond its usual circumscription to low-level motor processes. By tracking the maturation of coarticulatory organization, we can indeed capture the gradual binding of representational and executional levels. Expanding on that view, the present findings provide evidence for subtle differences in the implementation of this relationship due to the very nature of the phonemes represented in children’s mind and their motor expressions. From our preceding studies (Noiray et al., 2013, 2018, 2019; Rubertus and Noiray, 2018) and research conducted in the domains of lexical and phonological development, it seems that holistic and segmental organizations (both in representation and production) develop together, albeit probably at different paces at different times. For instance, lexically based organizations may prevail at an early stage because they support object-word correspondences and referencing which are particularly relevant for children at an early stage of their life, while segmental representations may develop more slowly because they are more abstract and not bound to real-world objects. While variability in individual trajectories is evidently to be expected (e.g., Smith et al., 2010), overall there is converging evidence in typically developing children that these types of organization integrate with one another in the course of developing spoken language fluency (e.g., Vihman, 2015).

Furthermore, we argue for an interactive approach to (spoken) language development in which various skills develop together and are equally important to the uniqueness of human communication. While the literature abounds with studies highlighting developmental interactions between phonological awareness and various cognitive domains (e.g. literacy: Ziegler and Goswami, 2005; or with vocabulary: Charles-Luce and Luce, 1995; Muter et al., 2004; Hilden, 2016), the present study sheds light on the interaction between cognitive and speech motor skills. Results suggest that motor, lexical, and phonological developments collaborate dynamically over time by contact with the language (i.e., *via* increasingly richer exposure and practice speaking the language). This is a fairly significant finding that has various implications.

First, it may challenge models of adult speech production that have suggested a modular approach with lexical, phonological, and motor processes considered as separate components sequentially orchestrated (e.g., Levelt and Wheeldon, 1994, Figure 1; Levelt, 1999, Figure 1). It may also promote a revision of speech production models that have considered interactions across domains but with a top-down approach, whereby motor execution depends on the output of preceding cognitive or neural processes (e.g., in Levelt and Wheeldon’s model: motor execution is comprised within phonological encoding but implemented as the final component, p. 245; in Guenther and Vladusich, 2012’s DIVA model: between the motor, auditory, and somatosensory domains, Figure 1, review in Tourville and Guenther, 2011). If interactions between the lexical, phonological, and motor domains exist in the developing speech system of children, those should prevail in adults’ speech organization or at least residuals from such relationships may remain. Assuming a developmental continuity from children to adults’ speech production, models of speech production would benefit in taking the ontogenetic findings into account and perhaps adopt a more integrated-interactive perspective. By doing so, it may be possible to move forward in the longstanding quest for determining the nature of the units of speech production (see, for example, discussion in Pierrehumbert, 2003; Hickok, 2014).

Second, the finding of interactions across domains is relevant for the clinical field. Indeed, while predictive studies have usually tested how skill *X* at a time T1 predicts the stage of another skill *Y* at time T2 (e.g., Walley et al., 2003; Edwards et al., 2004), no study has to our knowledge ventured to examine how interactions between specific skills change over developmental time or predict the stage of another interaction at a later time. Although the present study was not designed to demonstrate a specific causal direction in the relationships observed, it is highly likely that speech motor, lexical, and phonological skills mutually influence each other over time. There is enough evidence in infant and child research supporting both directions (e.g., motor, lexical and phonological developments: Menn and Butterworth, 1983; DePaolis et al., 2013; articulatory filter hypothesis: Vihman, 1996; DePaolis et al., 2011; Majorano et al., 2014; phonological templates: Vihman and Croft, 2007; Vihman and Wauquier, 2018; role of articulatory skills for later phonemic awareness). Given that coarticulated speech is initiated years before children gain adult-like knowledge about the structural combinatoriality of their native language, an effect of coarticulatory practice on the development of phonological awareness is not an implausible scenario. In the first 4-to-5 years of life, children acquire a basic awareness of the structural combinatoriality of *sounds* (phonetic awareness) because they can form new words (real words or imaginary creations) and converse comfortably with others. This raises the question whether phonological awareness is indispensable to adult-like fluent speech or only to fluent reading. To elucidate whether it is only a by-product of literacy acquisition that happens to create collateral changes to children’s speech organization, it will be crucial to examine whether the maturational trajectories of illiterate adults or children’s coarticulatory patterns are similar to those of literate children. If they do, it may suggest that developing adult-like coarticulatory patterns does not entail any advanced awareness of the structural combinatoriality of their native language. Instead, maturation of coarticulatory patterns may relate more to children tuning their speech motor system to the phonetic regularities of their native language and therefore interact more significantly with perceptual rather than phonological development. Expanding on this hypothesis, the process of language acquisition may encompass two types of interactions: one serving oral communication and primarily involving perceptual, motor, and lexical skills; another one developing in a more protracted fashion for the purpose of literacy acquisition and involving primary interactions between motor, lexical, and phonological skills. Comparisons with preschool-aged children with advanced phonemic awareness would also provide a compelling experimental framework for assessing the role of phonological awareness with respect to speech motor control skill for developing adult-like patterns of coarticulation. In a recently funded project, we have initiated a first step in this direction, testing for interactions between various levels of phonological awareness, reading proficiency, and production fluency in typically developing school-aged children (Popescu and Noiray, 2019) in comparison to children at risk or diagnosed with reading disorders.

### Limitations and Perspectives for Future Research

Overall, results from the present study provide strong evidence that the process of developing spoken language fluency encompasses dynamic interactions between vocabulary, phonological awareness, and speech motor control in German children. While this represents a promising first step, further empirical work is obviously needed to understand these multidimensional interactions in greater detail. Generalized additive modeling (GAM) represents an innovative and powerful method because it can unveil nonlinear relationships between cognitive and motor domains and estimate their interrelated change over time. In the present study, it was possible to use GAM models to illuminate nonlinear patterns of interactions, which would have remained hidden if we had used linear mixed models. Note, however, our dataset presents some weaknesses. For instance, the examination of vocabulary being limited to nouns in this study, our assessment of children’s expressive lexicon was limited, and hence, correlation should be considered with caution. As mentioned earlier, it was not possible to reliably test for the combined effect of vocabulary together with phonological awareness on coarticulatory coarticulation due to dataset requirements (e.g., recording many more children and obtaining many more scores per participant). For generalized additive modeling to provide reliable results, large sample-sized investigations are also necessary, which remain challenging in the developmental field due to various methodological constraints and time-consuming data processing. However, given the growing statistical expertise among developmental psycholinguists combined with greater effort to conduct synergistic data collection across laboratories, there is no doubt that future quantitative studies will succeed in teasing apart their (in)dependent effect on the development of spoken language fluency.

The present study is part of a longer-term project aiming to elucidate whether the expansion of vocabulary and phonological awareness contributes to increasingly more segmentally specified coarticulatory organizations from kindergarten to primary school. This question is not only important for theories of language acquisition but also for clinical practice. Assessments of deviant coarticulatory patterns have primarily tested their motor origins (e.g., apraxia of speech: Nijland et al., 2002; speech sound disorder: Maas and Mailend, 2012; phonological disorders: Gibbon, 1999; stuttering: Lenoci and Ricci, 2018). Evidence of an intricate relationship with other linguistic components of the language system would certainly affect the way diagnosis and treatment are envisioned. The opposite question whether increased practice coarticulating a wide range of phonetic combinations supports greater phonemic differentiation and the stabilization of motor correspondences would be equally exciting in terms of its implications for language-related cognitive development. In this study, we have first demonstrated that important interactions between cognitive and motor domains occur in the course of developing spoken language fluency. We believe our findings now warrant longitudinal investigations to further test whether the interactions observed are bi-directional and hence fundamental to the growth of each individual skill or unilateral.

Last, if phonological awareness is the knowledge of the discrete and coarticulation represents its continuous articulatory-acoustic make-up, it will be important in future studies to design analytical approaches that can adequately account for the development of this intricate relationship over time. Dynamical systems seem a promising avenue in that respect. In a recent discussion of speech dynamics, Iskarous emphasizes that dynamical systems “do not assume separate sets of principles to describe discrete and continuous aspects of a system. Rather, the discrete description is shown to predict the continuous one, using the concept of a differential equation” (Iskarous, 2017, p. 8). The present study provides an ontogenetic perspective illustrating how access to various levels of phonological discreteness (words, syllables, segments) interacts with the organization of the continuous: from the production of syllabic entities to the fine integration of segmentally specified gestures. In future research on this topic, we aim to combine dynamical systems theory with longitudinal data to address how this dynamical relationship precisely unfold in the developing language system of children.

## Conclusion

The present study tested whether developmental differences in coarticulation degree widely reported in the literature over the past decades were strictly related to maturational differences in speech motor abilities or also interacted with children’s language-related abilities. An examination of children’s coarticulatory patterns in relation to their lexical and phonological proficiency allowed us to uncover developmental differences that would remain unexplained if each skill was considered separately. Other domains, which have not been examined in the present study, are likely to play a role and should be thoroughly considered in future studies (e.g., assessment of literacy, phonological memory). The question of what skill interactions allow children to become fluent language users and how those evolve dynamically over time have become pressing issues for developmental researchers. However, for those to be uncovered interdisciplinary collaborations will be necessary, between developmental biology, psychology, and linguistics. While all domains have separately argued that multiple developments are intricately connected over time, only actual collaborations across disciplines will generate a unified account of language development.

## Data Availability Statement

The datasets generated for this study are available on request to the corresponding author.

## Ethics Statement

The study reported in the manuscript has been approved by the Ethic Committee of the University of Potsdam in Germany. The goals of the research, the children population recorded, the method, and recruitment procedure have been described and reviewed by the Committee prior to giving a positive review.

## Author Contributions

AN provided the theoretical framework of the study, obtained the funding, and designed the empirical questions resulting in the manuscript. AN and AP conceptualized and designed the statistical analyses. AN, AP, and LH organized the dataset for subsequent statistical analyses. AP performed all statistical analyses. AN, AP, HK, ER, SK, and LH contributed to ultrasound data collection and processing and/or administration and scoring of the behavioural assessments. HK trained the team in administration and scoring the developmental assessments. AN wrote the manuscript. AN and AP provided all visualizations and edited the first draft. HK, ER, and SK provided feedback on the pre-final draft. All authors read the manuscript and agreed on its submission.

### Conflict of Interest

The authors declare that the research was conducted in the absence of any commercial or financial relationships that could be construed as a potential conflict of interest.
